# Supplementary material for: Preclinical usability evaluation of the Liveborn app: A mobile health application that provides feedback for neonatal resuscitation
Source: PLOS Digit Health. 2025 Apr 23;4(4):e0000814. doi: 10.1371/journal.pdig.0000814 (PMC12017530; doi:10.1371/journal.pdig.0000814)
Supplement: S1 Table — (DOCX) [file pdig.0000814.s001.docx]

**S1 Table.** Simulation design targeting common resuscitation errors and a variety of app features

|  | **Common Errors Targeted** | **App Feedback Targeted** |
| --- | --- | --- |
| **Real-Time Guidance Simulations** | | |
| **Case 1: Cry, needs stimulation**  **(Yellow Zone)^1^** | Lots of suctioning | Time prompts  Avoid prolonged suctioning |
| **Case 2: Apneic at birth**  **(Red Zone)** | Delayed BMV  Lots of suctioning | Place NeoBeat  Ventilate non-breathing babies  Avoid prolonged suctioning |
| **Case 3: Cries 🡪 apnea**  **(Red Zone)** | Delayed BMV | Place NeoBeat  Evaluate breathing  Ventilate non-breathing babies |
| **Case 4: BMV, low heart rate**  **(Red Zone)** | Ineffective BMV | Ventilate non-breathing babies  Improve ventilation  Seek advanced care |
| **Case 5:** **Fresh stillbirth** | No attempt to resuscitate stillborn | Dry/stimulate shortly after birth  Ventilate non-breathing babies  Improve ventilation  Evaluate heart rate and breathing |
| **Debriefing Simulations** | | |
| **Case 1: Cries at birth**  **(Green Zone**) | No skin-to-skin  Early cord clamping | Stimulation: ok  Skin-to-skin: none OR paused  Cord clamp: too early  Suction: none OR registered  Baby condition: well |
| **Case 2: Cry, needs stimulation**  **(Yellow Zone)** | Lots of suctioning | NeoBeat: ok  Stimulation: ok  Skin-to-skin: registered (OR none)  Cord clamp: ok  Suction: excessive  Baby condition: well |
| **Case 3: Needs BMV**  **(Red Zone)** | Prolonged stimulation  Prolonged suction  Delayed BMV | NeoBeat: delayed  Stimulation: prolonged  Suction: prolonged  BMV time: delayed  BMV continuous: ok (OR paused)  Baby condition: well |
| **Case 4: BMV but dies**  **(Red Zone)** | Ineffective BMV | NeoBeat: ok  Stimulation: ok (OR none)  Suction: registered  BMV time: ok (OR delayed)  BMV cont: ok (OR paused)  Baby condition: dead |
| **Case 5 Fresh stillbirth** | No attempt to resuscitate stillborn | NeoBeat: ok  Stimulation: ok (OR none)  Suction: none  BMV time: none  Baby condition: dead |
| Abbreviation: BMV=bag-mask ventilation  ^1^Zones reflect the Helping Babies Breathe action plan with Green referring to babies who cry at birth, Yellow referring to babies who need stimulation to breathe and Red referring to babies who need BMV. | | |
