# Supplementary material for: Preclinical usability evaluation of the Liveborn app: A mobile health application that provides feedback for neonatal resuscitation
Source: PLOS Digit Health. 2025 Apr 23;4(4):e0000814. doi: 10.1371/journal.pdig.0000814 (PMC12017530; doi:10.1371/journal.pdig.0000814)
Supplement: Attachment — Submitted filename: Response to Reviewers PLOS Digital Health.pdf [file pdig.0000814.s003.pdf]

UNC SCHOOL OF MEDICINE

UNC HEALTH Children's

Division of Neonatal-Perinatal Medicine
The University of North Carolina at Chapel Hill
UNC Hospitals, CB# 7596, 4th Floor, Rm N4051
Chapel Hill, NC 27599-7596
Office: 984-974-5063 • Fax: 984-974-7857

February 27, 2025

Leo Anthony Celi
Editor-in-Chief
PLOS Digital Health

Dear Dr. Celi,

Thank you for your further consideration of our manuscript, now titled "*Preclinical Usability Evaluation of the Liveborn App: a Mobile Health Application that Provides Feedback for Newborn Resuscitation" (PDIG-D-24-00572)*. We thank you and the reviewers for their helpful comments, and respectfully submit the final revised manuscript. Below is a point-by-point response to the reviewers' comments.

Sincerely,

Jackie K. Patterson, MD, MPH
Associate Professor of Pediatrics
Division of Neonatal-Perinatal Medicine
University of North Carolina at Chapel Hill

Reviewer #1

I highly value the development effort on the liveborn app, as this is likely impactful in both LMIC setting as well as elsewhere. I have read this paper with a background on perinatal clinical research, with some, but limited experience on app development in clinical care. The specific value hereby relates to the integration with eg HBB construct.

1) Post hoc, I perceive the title somewhat misleading, as the focus is rather on preclinical (simulations) product optimization and development, not similar to usability evaluation (as this suggests in my perception clinical use). In my opinion, the usability in real life (efficacy versus effectiveness) will likely have additional issues like manpower – second pair of hands needed, resources, educational level needed etc. The tool is likely rather ready to assess usability and feasibility in real life?

   Response: The title may indeed suggest a focus on clinical usability evaluation rather than on product optimization through simulations. We acknowledge this distinction and have clarified the title to explicitly reflect that this study assesses usability in simulated settings—an essential step before real-world clinical evaluation. The new title is: "*Preclinical Usability Evaluation of the Liveborn App: a Mobile Health Application that Provides Feedback for Newborn Resuscitation."* We prefer to maintain "usability" in the title as literature on evaluation of mobile health tools commonly uses this term to refer to the type of evaluation we conducted (see Maramba et al., Methods of usability testing in the development of eHealth applications: A scoping review. Int J Med Inform 2019 June: 126: 95-104).

2) In your 'lay summary', you rightfully use failure to breathe at birth, and this description is in my opinion more accurate than the 'respiratory depression' in the abstract.

   Response: We agree that "failure to breathe at birth" is a more readily understood than the term "respiratory depression." We chose respiratory depression due to increasing use of this term over that of birth asphyxia as respiratory depression focuses on the symptom without attributing a particular etiology (see Lee et al., Intrapartum-related neonatal encephalopathy incidence and impairment at regional and global levels for 2010 with trends from 1990. Pediatr Res. 2013 Dec 20; 74(Suppl 1): 50-72). That said, given the audience for this manuscript, we appreciate your suggestion and have altered our terminology to "failure to breathe at birth" throughout the manuscript, including in the abstract.

3) Were the same or other midwives recruited for the first and second round of the app?

   Response: The same midwives participated in both rounds of the evaluation. This methodological choice ensures a reliable assessment of usability improvements and avoids introducing variability due to differences in familiarity with the tool.

4) Why 10 simulations (2 x 5) per round. Was this based on any 'power', or rather pragmatic?

Response: Our decision to conduct 10 simulations per round for two rounds was a programmatic decision. We made this decision based on our experience to date with the app including the types of usability problems we anticipated, the highly structured task we were testing, and the homogeneity of participants. We have added these details to the methods section under study procedures.

Reviewer #2

In their manuscript, Ishoso et al. describe a usability study of the Liveborn app, which was carried out in the Democratic Republic of Congo. The goal of Liveborn is to improve neonatal outcomes by providing real-time feedback and debriefing for neonatal resuscitation in low-resource settings. The overall Liveborn project has the potential to meaningfully improve global health, and I look forward to reading the results from their ongoing cluster randomised trial.

Overall, the manuscript is clear and well-written, and the authors should be commended for readily discussing both the strengths and the limitations of their usability study. The work highlights the importance of iterative design in mHealth applications. I have no major concerns but I do have a number of questions and minor comments which I wish were addressed in the text.

1) Data sharing

There are significant commercial implications of this work, which is presumably why the manuscript does not describe the interface of the Liveborn app in detail. This is understandable, but when the authors state that “all data files will be available …”, what data is this referring to?

Response: All data underlying the findings presented in the results section of this manuscript will be freely available in a public repository upon publication, in keeping with the PLOS Data policy.

2) Languages

The French version of Liveborn is used in this study. What other languages is Liveborn available in? Additionally, how does the team think about and ensure quality of translation?

Response: Liveborn is currently available in French, the primary language of healthcare professionals in the DRC. Recognizing the importance of accurate translation, we collaborated with local clinicians to ensure appropriate terminology. Additional language versions may be considered based on future deployment needs. To address these questions, we added the following sentence to the methods:

> “We used a French version of Liveborn in this usability study as French is the primary language of professional healthcare workers in the DRC; the translation was verified by

> local clinicians to ensure accuracy and appropriateness." (Methods, Description of the Liveborn app, Lines 132-135)

3) Guidelines used

What are the resuscitation guidelines / clinical algorithms underpinning Liveborn? The authors mention Helping Babies Breathe (L84), and I understand this is based on ILCOR guidelines. However, I can imagine that in different hospitals, slightly different resuscitation algorithms may be used. Can the app be customised to take this into account? How likely is it that the app recommends action that is different from established practice at the site it is used in?

Response: Liveborn was designed for low-resource settings; since Helping Babies Breathe (HBB) is the most commonly used algorithm in these settings, we based its feedback on HBB. That said, the feedback provided by Liveborn is generally consistent with resuscitation practices that are widely endorsed across a variety of protocols in low-resource settings, and aligns with ILCOR guidelines—for example, recommending initiation of ventilation within the golden minute, and recommending corrective steps to face mask ventilation if the infant's clinical condition is not improving. As such, although resuscitation protocols may vary across hospitals, we expect the feedback from Liveborn will be generally applicable across a variety of low-resource settings.

The app is not currently customizable to take into account different protocols. However, we are continuing to refine the feedback provided by the app based on our clinical pilot and an on-going randomized trial.

To the extent that standard practices at a site align with evidence-based resuscitation practices, the app's recommendations will not differ from established practice. In cases where standard practice at a facility does not follow the evidence—such as habitual and prolonged suctioning of neonates at the expense of delaying ventilation—the app will recommend actions that differ from common practice at that facility.

To summarize the above details, we added the following sentence to the methods:

> "The feedback given by Liveborn aligns with recommendations from the International Liaison Committee on Resuscitation (ILCOR) and is specifically based on the HBB algorithm." (Methods, Description of the Liveborn app, Lines 130-132)

5) Integration with NeoBeat

It is not immediately clear whether NeoBeat is necessary for use of the Liveborn app. Can Liveborn be used without continuous heart rate monitoring of the neonate? Or using another heart rate monitoring device? I imagine that in a low-resource setting, neonatal heart rate monitoring is not always available.

Response: Yes, Liveborn can function without continuous heart rate monitoring via NeoBeat. While NeoBeat provides objective heart rate data to inform feedback from Liveborn, the app can still provide feedback relying solely on manual event recording by an observer. There is no current functionality within Liveborn to allow for integration with a different heart rate monitoring device. We have added the following sentence to the methods:

> "While Liveborn can provide feedback relying solely on data from the observer, the objective heart rate data from NeoBeat allows for more nuanced feedback." (Methods, Description of the Liveborn app, Lines 125-127)

6) Paired work

During both arms of the usability study (real-time feedback and debrief), midwives worked in pairs. Is the intention that Liveborn is used like this in the clinic, and how feasible is it that every time Liveborn is used in the clinic, a qualified midwife or nurse would be available as an observer who records data and interacts with the app?

Response: In our study, midwives worked in pairs (one performing resuscitation and another documenting events). In clinical practice, we acknowledge that always having a second qualified staff member available may be challenging. In a clinical pilot, we have explored alternative strategies to identify observers in a health facility including training nursing students and environmental health services staff. We recognizing the need for an observer is a significant limitation to scale-up of this tool, and are exploring strategies for automated data collection including the use of video with AI interpretation of events.

7) SUS improvement

Is the System Usability Score improvement between rounds 1 and 2 statistically significant?

Response: We have now tested for statistically significant changes of the SUS and FIM scores between rounds. The SUS improvement between rounds was not statistically significant. The additions/changes to the methods and results are as follows:

> "To evaluate the statistical significance of changes in scores between rounds for both the SUS and FIM, we used the Wilcoxon signed-rank test for paired samples." (Methods, Outcomes and Analysis, Lines 187-189)

> "The median usability score measured by the SUS was 81 (77, 86) in Round 1 and 90 (85, 95) in Round 2 (p=0.23; Fig 3). The median feasibility score measured by the FIM was 18 (18, 19) in Round 1 and 19 (16, 20) in Round 2 (p=0.94; Fig 4)." (Results, Usability and Feasibility, Lines 262-264)

7) Participant demographics
What was the reason participants were overwhelmingly older and universally very experienced

/ why were more junior midwives not included in the study? If the goal of Liveborn is to improve adherence to resuscitation guidelines, feedback from more junior clinicians would be essential.

Response: Participants were selected from the existing workforce at the partner facility, all of whom had over 10 years of clinical experience and were relatively inexperienced with apps. While we acknowledge that involving junior midwives could have provided additional insights, we think this will be particularly important for clinical evaluations when we are evaluating adherence to guidelines.

8) Inaccuracies in recording
Even after Round 2, authors found inaccuracies in data recording persisted. They suggest further training could improve this (L234-236), which I am somewhat skeptical of. How common and how problematic is the issue of inaccuracy with the updated app interface?

Response: We have several years of experience with the data collection function of the Liveborn app collecting clinical data during resuscitations in health facilities in Kinshasa, DRC. It has been our experience that accuracy of data collection improves over time with additional training and repeated use. To enhance accuracy, we have engaged in coaching of observers including practice recording data using resuscitation videos. We acknowledge the training and practice required to support highly accurate data collection is a barrier to use. We are engaged in on-going research to compare the accuracy of observer-gathered data with Liveborn to a gold standard of video recording. Although we describe inaccuracies in data recording during this simulation pilot, we think these inaccuracies are not profound in light of the current standard of documenting resuscitation events via recall.

9) Figure quality
Please not figure resolution is not sufficient for full-page viewing.

Response: We have ensured the figure resolution meets journal specifications.
